# MicroRNA, *hsa-miR-200c*, is an independent prognostic factor in pancreatic cancer and its upregulation inhibits pancreatic cancer invasion but increases cell proliferation

**DOI:** 10.1186/1476-4598-9-169

**Published:** 2010-06-28

**Authors:** Jun Yu, Kenoki Ohuchida, Kazuhiro Mizumoto, Norihiro Sato, Tadashi Kayashima, Hayato Fujita, Kouhei Nakata, Masao Tanaka

**Affiliations:** 1 Department of Surgery and Oncology, Graduate School of Medical Sciences, Kyushu University, Fukuoka, Japan; 2 Department of Pathology, The Sol Goldman Pancreatic Cancer Research Center, The Johns Hopkins Medical Institutions, Baltimore, MD, USA; 3 Department of Advanced Medical Initiatives, Graduate School of Medical Sciences, Kyushu University, Fukuoka, Japan; 4 Kyushu University Hospital Cancer Center, Fukuoka, Japan; 5 Department of Surgery, Japanese Red Cross Fukuoka Hospital, Fukuoka, Japan

## Abstract

**Background:**

Recently, the microRNA-200 family was reported to affect cancer biology by regulating epithelial to mesenchymal transition (EMT). Especially, the expression of *miR-200c *has been shown to be associated with upregulating the expression of *E-cadherin*, a gene known to be involved in pancreatic cancer behavior. However, the significance of *miR-200c *in pancreatic cancer is unknown.

**Methods:**

In the present study, we investigated the relationship between *E-cadherin *and *miR-200c *expression in a panel of 14 pancreatic cancer cell lines and in macro-dissected formalin-fixed paraffin-embedded (FFPE) tissue samples obtained from 99 patients who underwent pancreatectomy for pancreatic cancer. We also investigated the effects of *miR-200c *on the proliferation and invasion of pancreatic cancer cells.

**Results:**

We found that patients with high levels of *miR-200c *expression had significantly better survival rates than those with low levels of *miR-200c *expression. We also found a remarkably strong correlation between the levels of *miR-200c *and *E-cadherin *expression.

**Conclusions:**

These data indicate that *miR-200c *may play a role in the pancreatic cancer biology and may be a novel marker for the prognosis of pancreatic cancer.

## Introduction

Pancreatic cancer is the fifth leading cause of cancer death and has the lowest survival rate of any solid cancer in the industrialized countries [1,2]. In the past 20 years, 6942 Japanese patients with pancreatic cancer who underwent pancreatectomy showed a very poor prognosis with an overall median survival time (MST) of 11.7 months and a 5-year survival rate of 13.4% [1]. Extensive molecular analysis of pancreatic cancer has led to discoveries of genetic, epigenetic and, more recently, microRNA alterations [3-6].

MicroRNAs (miRNAs) are endogenous, small non-coding RNAs of 14-24 nucleotides that can negatively regulate protein expression at the post-transcriptional level by translational inhibition and/or mRNA degradation, mostly through base pairing with the 3'-UTR of their target mRNAs [7]. Recently, the abnormal expression of miRNAs was shown to be correlated with cancer. The first evidence suggesting a direct link between miRNAs and human cancer was the localization of *miR-15a *and *miR-16-1 *within a 30 kb region of minimal loss on chromosome 13 that is deleted in chronic lymphocytic leukemia (CLL) and that both genes are often deleted or down-regulated in CLL [8]. Other miRNAs, such as *miR-143 *and *miR-145 *have reduced levels of expression in adenomatous and cancerous stages of colorectal neoplasia [9], while *let-7 *expression is reduced in lung tumor [10]. The first oncogenic miRNAs (oncomiR-1), the miR-17-92 cluster, named from the research on human B cell lymphomas [11], were described as tumor suppressors or oncogenes and brought a novel area of investigation to cancer research [12,13].

Recently, it has been reported that *miR-200c *is a marker of aggressiveness and chemoresistance in female reproductive cancers, that *miR-200c *suppresses invasiveness and restores sensitivity to microtubule-targeting chemotherautic agents in breast and ovrian cancer cells, and that downregulation of *miR-200c *links breast cancer stem cells with normal stem cells [14-16]. Meanwhile, Hurteau *et al*. revealed that over-expression of *miR-200c *leads to reduced expression of *transcription factor 8 *(*TCF8*; also termed *ZEB1*) and increased expression of *E-cadherin *in breast cancer cells [17,18]. Also, Park *et al*. reported that *miR-200c *regulates epithelial to mesenchymal transition (EMT) and restores expression of *E-cadherin *in breast and ovarian cancer cells [18-20]. EMT is a central process in the progression of primary tumors toward metastasis (a switch from the polarized, epithelial phenotype to a highly motile fibroblastoid or mesenchymal phenotype). Furthermore, expression of *E-cadherin *can predict disease outcome in patients with resectable pancreatic carcinoma, and the therapeutic restoration of *E-cadherin *was proposed as a strategy to suppress cancer metastasis [21-24].

In the present study, to identify novel relationship between *E-cadherin *and *miR-200c *in pancreatic cancer, we quantified *miR-200c *expression in a panel of 14 pancreatic cancer cell lines and in 99 samples of macro-dissected formalin-fixed paraffin-embedded (FFPE) pancreatic tissues. We also investigated the *in vitro *effects of *miR-200c *upregulation on the proliferation and invasion of pancreatic cancer cells. We found that patients with high levels of *miR-200c *expression had significantly better survival rates compared to those with low levels of *miR-200c *expression. We also found striking correlation between with the levels of *miR-200c *and *E-cadherin *expression. These data suggest that *miR-200c *may be a novel marker for the prognosis of pancreatic cancer.

## Materials and methods

### Cultured cells

The following 15 pancreatic cancer cell lines were studied: AsPC-1, KP-1N, KP-2, KP-3, PANC-1, BxPC-3 and SUIT-2 (provided by Dr. H. Iguchi, National Shikoku Cancer Center, Matsuyama, Japan); MIA PaCa-2 (Japanese Cancer Resource Bank, Tokyo, Japan); NOR-P1 (established in our laboratory by Dr. Sato); CAPAN-1, CAPAN-2, CFPAC-1, H48N, HS766T and SW1990 (American Type Culture Collection, Manassas, VA, USA). In addition, a human pancreatic ductal epithelial cell line (HPDE, provided by Dr. Ming-Sound Tsao, University of Toronto, Toronto, Ontario, Canada) was studied. The cells were maintained as described previously [25].

### Pancreatic tissues

Our study consisted of 99 patients who underwent pancreatic resection for pancreas cancer at the Department of Surgery and Oncology, Kyushu University Hospital (Fukuoka, Japan) from 1992 to 2007. The patients comprised 64 men and 35 women with a median age of 66 years (range, 36-86 years). Survival was measured from the time of pancreatic resection and death was the endpoint. Prognosis was examined in October 2008. The median observation time for overall survival was 15 months and it ranged from 1 to 101 months. Sixty four patients died during follow-up and the other patients were alive and censored.

All resected specimens were fixed in formalin and embedded in paraffin (FFPE) for pathological diagnosis. All tissues adjacent to the specimens were evaluated histologically according to the criteria of the World Health Organization. For all cases, two pathologists were in agreement with regard to pathological features and both confirmed the diagnoses. The stage of tumors was assessed according to the Union Internationale Contre le Cancer (UICC) classification. The clinicopathological characteristics of the tumor collection are described in Table 1. Written informed consent was obtained from all patients, and the study was approved by the Ethics Committee of Kyushu University and conducted according to the Ethical Guidelines for Human Genome/Gene Research enacted by the Japanese Government and the Helsinki Declaration.

**Table 1:** Clinicopathological Characteristics of 99 Patients with Pancreatic Cancer

Median age			65.7 years (range, 36-86 years)	
Sex (Male/Female)			62 (62.6%)/37 (37.4%)	
Histological diagnosis				
Adenocarcinoma			97 (98.0%)	
Adenosquamous carcinoma			2 (2.0%)	
pT category				
pT1			6 (6.1%)	
pT2			3 (3.0%)	
pT3			57 (57.6%)	
pT4			33 (33.3%)	
pN category				
pN0			33 (33.3%)	
pN1			66 (66.7%)	
UICC stage				
IA & IB			5 (5.1%) & 4 (4.0%)	
IIA & IIB			21 (21.2%) & 64 (64.7%)	
III			1 (1.0%)	
IV			4 (4.0%)	
Histological grade				
G1			20 (20.2%)	
G2			43 (43.4%)	
G3			36 (36.4%)	
Residual tumor category				
R0			60 (61.2%)	
R1			38 (38.7%)	
Vessel invasion				
Positive			61 (62.2%)	
Negative			37 (37.7%)	
Neural invasion				
Positive			84 (84.8%)	
Negative			15 (15.2%)	

### miRNA isolation

miRNAs were extracted from cultured cells using a *mir*Vana™ miRNA Isolation Kit (Ambion, Austin, TX, USA) and from macro-dissected FFPE pancreatic tissues using an RNeasy^® ^FFPE Kit (Qiagen, Tokyo, Japan), following the manufacturer's instructions. Considering the influence of genomic DNA contamination, especially from the FFPE materials, Qiagen provides a special gDNA Eliminator spin column to rapidly remove genomic DNA, and we also performed a DNase digestion step. The extracted RNA was quantified by absorbance at 260 nm and its purity was evaluated by the absorbance ratio at 260/280 nm with a NanoDrop ND-1000 spectrophotometer (NanoDrop Technologies, Rockland, DE, USA).

### Quantitative real-time reverse-transcription polymerase chain reaction (qRT-PCR)

The expression of *miR-200c *and *RNU6B *(*U6 *snRNA, a reference gene) was measured by qRT-PCR using a TaqMan^® ^MicroRNA Reverse Transcription Kit and TaqMan^® ^Universal PCR Master Mix (No AmpErase^® ^UNG; Applied Biosystems, Tokyo, Japan) and a Chromo4™ System (Bio-Rad, Hercules, CA, USA). We followed the manufacturer's protocols to perform two-step real-time RT-PCR for the measurement of *miR-200c *and *RNU6B *expression. Each sample was run in triplicate. The level of *miR-200c *expression was calculated from a standard curve constructed with small RNAs from the CAPAN-1 pancreatic cancer cell line. The expression levels of *miR-200c *were normalized against the corresponding expression levels of *RNU6B*.

The levels of *E-cadherin *mRNA and *18S *rRNA were measured by qRT-PCR using a QuantiTect SYBR Green RT-PCR Kit (Qiagen, Tokyo, Japan) and a Chromo4™ System, following the manufacturer's protocols [26]. Each sample was run in triplicate. We designed specific primers for *E-cadherin *(forward, 5'-tcagcgtgtgtgactgtgaa-3'; reverse, 5'-aggctgtgccttcctacaga-3'), and *18S *rRNA (forward, 5'-ctttcgaggccctgtaattg-3'; reverse, 5'-cctccaatggatcctcgtta-3') using Primer 3 software and performed BLAST searches to ensure the specificity of the primers. The PCR products amplified using these primers are small (*18S rRNA*, 63 bp; *E-cadherin*, 53 bp), which allowed accurate and sensitive qRT-PCR despite the fragmented RNA extracted from FFPE tissue specimens [27,28]. We also included controls without reverse transcriptase to confirm that there was no influence from genomic DNA contamination. The level of *E-cadherin *mRNA was calculated from a standard curve constructed with total RNA from CAPAN-1 cells and normalized against levels of *18S *rRNA. Accuracy and integrity of PCR products were confirmed with an Agilent 1000 Bioanalyzer (Agilent Technologies, Palo Alto, CA, USA).

### Cell transfection with miRNA precursors

Upregulation of *miR-200c *expression was achieved by transfection with the *hsa-miR-200c *precursor (Pre-miR™ miRNA Precursor; Applied Biosystems). To verify the specificity of the transfection effect, we used a Pre-miR™ miRNA Precursor Negative Control (Applied Biosystems). Transfections were performed by electroporation using a Nucleofector system (Amaxa Biosystems, Köln, Germany) according to the manufacturer's instructions. PANC-1, SUIT-2 and KP-2 cells (1-2 × 10^6^) were transfected with 100 pmol of the indicated precursor or negative control. The degree of mature *miR-200c *upregulation 48 h after transfection was verified by quantifying the expression level of mature *miR-200c*. Cells harvested 48 h after transfection were also used for cell proliferation or invasion assays.

### Propidium iodide (PI) assay

Cell proliferation was evaluated using a multiwell fluorescence plate reader and a previously described method [29] with modifications [30,31]. Briefly, cancer cells were seeded at 2 × 10^4 ^cells/well in Falcon flat-bottom 24-well plates (Becton Dickinson, Franklin Lakes, NJ, USA). 30 μM PI (Wako Ltd., Osaka, Japan) and 600 μM digitonin (Wako Ltd.) were then added to each well. After incubation for 90 min of at 37°C, the fluorescence intensities of labeled nuclei were measured using a CYTO Fluor™ II fluorescence multiwell plate reader (PerSeptive Biosystems, Framingham, MA, USA) to determine total cell numbers.

### *In vitro *Matrigel invasion assay

Invasion of pancreatic cancer cells was evaluated by the numbers of cells invading Matrigel-coated transwell inserts (Becton Dickinson) as reported previously [25,32]. Briefly, transwell inserts with 8 μm pores were coated with Matrigel (20 μg/well; Becton Dickinson). Cancer cells were seeded in the upper chamber at a density of 1.0 × 10^5 ^cells/cm^2 ^in 250 μl of Dulbecco's modified Eagle's medium (DMEM) supplemented with 10% fetal bovine serum (FBS). After incubation at 37°C, cells that had invaded to the lower surface of the Matrigel-coated membranes were fixed with 70% ethanol, stained with hematoxylin and eosin (H & E) and counted in five randomly selected fields under a light microscope.

### Statistical analysis

The *in vitro*data are presented as mean values with error bars representing the minimum and maximum or with the standard deviation (SD). The significance level was *p *< 0.05. *MiR-200c *expression in macro-dissected FFPE samples was split into high and low expression groups using a recursive descent partition analysis. Categorical variables were compared with the chi-square test (Fisher's exact probability test). Survival curves were constructed with the Kaplan-Meier product-limit method and compared by log-rank tests. To evaluate independent prognostic factors associated with survival, a multivariate Cox proportional hazards regression analysis was used, with *miR-200c *expression, age, sex pathological tumor (pT) status, pathological node (pN) status, UICC stage, residual tumor (R) status, histological grade (G) and vessel invasion as covariates (Table 2). Statistical significance was defined as *p *< 0.05. The statistical analyses in the macro-dissected FFPE samples were performed with JMP 7.01 software (SAS Institute, Cary, NC, USA).

**Table 2:** Univariate and Multivariate Survival Analyses

	Univariate analysis			Multivariate analysis		
		
Characteristics	Hazard Ratio (HR)	95% confidence interval	*P *value	Hazard Ratio (HR)	95% confidence interval	*P *value
Age (< 65)	0.9	0.5 - 1.4	0.62	0.8	0.5 - 1.4	0.50
Sex(Female)	0.9	0.5 - 1.5	0.63	0.9	0.5 - 1.6	0.69
pT (pT1/2)	2.2	1.4 - 3.6	< 0.001	1.8	0.5 - 5.4	0.35
pN (negative)	0.4	0.2 - 0.7	< 0.001	0.5	0.3 - 1.0	0.06
UICC stage	-	-	0.003	-	-	0.01
Histological grade (G3)	1.7	0.9 - 2.8	0.07	0.8	0.6 - 2.6	0.8
Residual tumor (positive)	3.0	1.8 - 5.0	< 0.001	3.2	1.8 - 5.8	< 0.001
Vessel invasion (positive)	2.3	1.4 - 4.1	0.001	1.9	1.0 - 3.6	0.03
Low *miR-200c*	1.8	1.0 - 3.5	0.03	2.2	1.1 - 4.6	0.02

Relative risk of UICC stage was not shown because of 2 parameters.

## Results

### Quantitative analysis of *miR-200c *expression in pancreatic cancer cell lines

**Figure 1:**
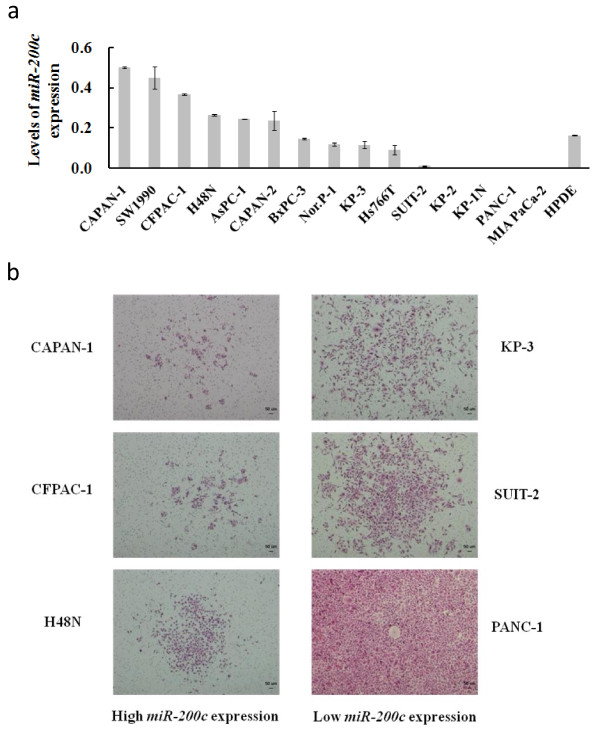
**The levels of *miR-200c *expression in cell lines and the correlation of *miR-200c *expression level and the invasion ability of pancreatic cancer cell lines**. a. The levels of *miR-200c *expression in 15 pancreatic cancer cell lines and in an HPDE cell line. b. Pictures of invading cells from cell lines expressing high levels of *miR-200c *(CAPAN-1, CFPAC-1 and H48N) and from cell lines expressing low levels of *miR-200c *(KP-3, SUIT-2 and PANC-1). H & E staining. Original magnification, 10 ×. Each sample was run in triplicate. Error bars represent SD.

We investigated *miR-200c *expression in 15 pancreatic cancer cell lines and in a non-neoplastic ductal epithelial cell line (HPDE) by quantitative real-time RT-PCR. As shown in Figure 1a, 4 pancreatic cancer cell lines, CAPAN-1, SW1990, CFPAC-1, and H48N, expressed higher levels of *miR-200c *than HPDE. Two pancreatic cancer cell lines, AsPC-1 and CAPAN-2, expressed similar levels of *miR-200c *to HPDE and 9 pancreatic cancer cell lines, BxPC-3, NOR-P1, KP-1N, KP-2, KP-3, Hs766T, SUIT-2, PANC-1 and MIA PaCa-2, expressed lower levels of *miR-200c *than HPDE.

### High levels of *miR-200c *expression correlated with low invasion ability

Having determined the levels of *miR-200c *expression in the 15 pancreatic cancer cell lines, we investigated the invasion ability of the cell lines that expressed high levels of *miR-200c *(CAPAN-1, CFPAC-1, and H48N) and of the cell lines that expressed low levels of *miR-200c *(KP-3, SUIT-2, and PANC-1) using the Matrigel invasion assay. We seeded 7.5 × 10^4 ^cells per Matrigel-coated well and counted the cells that had invaded the Matrigel 50 h after seeding. As shown in Figure 1b, all cell lines that expressed high levels of high *miR-200c *(CAPAN-1, CFPAC-1, and H48N) showed fewer numbers of invading cells compared to the cell lines that expressed low levels of low *miR-200c *(KP-3, SUIT-2, and PANC-1).

### Quantitative analysis of *E-cadherin *mRNA levels in cell lines and significant correlations between *miR-200c *and *E-cadherin *mRNA levels

**Figure 2:**
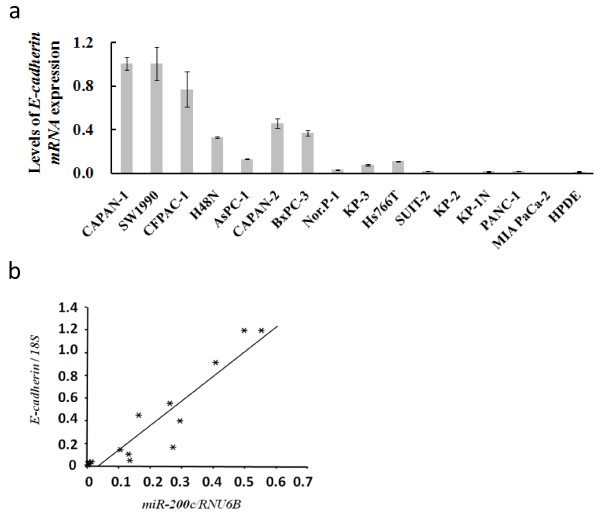
***E-cadherin *expression in cell lines and the correlation between *E-cadherin *and *miR-200c *expression**. a. The levels of *miR-200c *expression in 15 pancreatic cancer cell lines and in an HPDE cell line. b. The correlation between *miR-200c *and *E-cadherin *mRNA levels in all cell lines (*p *< 0.0001). Each sample was run in triplicate. Error bars represent SD.

We investigated *E-cadherin *mRNA levels by qRT-PCR in the 15 pancreatic cancer cell lines and in the HPDE cell line. Similar to the results of *miR-200c *expression, there were high or low *E-cadherin *mRNA levels in these cell lines (Figure 2a), and we found there were significant correlations between *miR-200c *and *E-cadherin *mRNA levels in all cell lines (Pearson's test *p *< 0.0001, Figure 2b)

### Upregulation of *miR-200c *in pancreatic cancer cell lines

**Figure 3:**
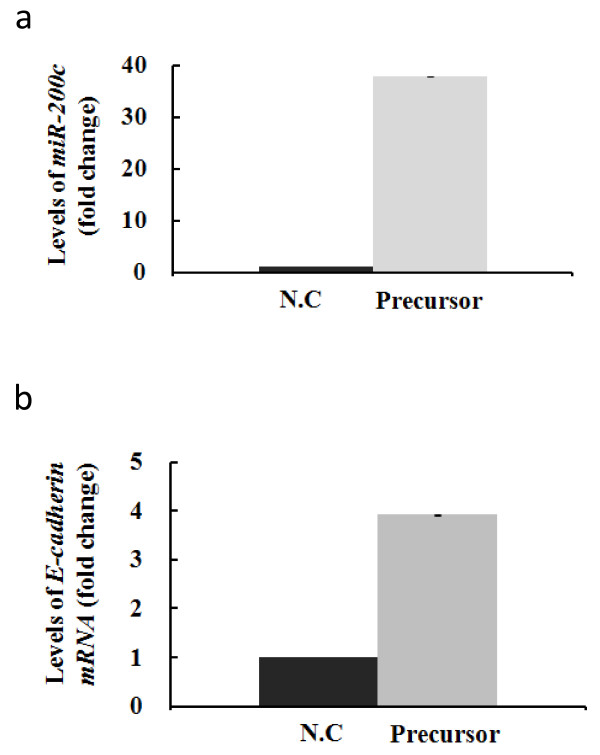
**Upregulation of *miR-200c *in pancreatic cancer cell lines and enhanced expression of *E-cadherin***. a. SUIT-2 cells were transfected with *hsa-miR-200c *precursor and showed a 38-fold increase in mature *miR-200c *expression compared with the control 24 h after transfection. b. Upregulation of *miR-200c *enhanced expression of *E-cadherin *3.9-fold relative to the control group. Each sample was run in triplicate. Error bars represent SD.

To upregulate the expression of mature *miR-200c*, we transfected the pancreatic cancer cell lines that expressed *miR-200c *at low levels with the *miR-200c *precursor. 24 h after transfection, we isolated total RNA (including small RNAs) and investigated the levels of *miR-200c *expression. As shown in Figure 3a, SUIT-2 cells transfected with the hsa-*miR-200c *precursor (precursor group) showed a 38-fold increase in mature *miR-200c *expression compared with cells transfected with the miRNA Precursor Negative Control (control group). Similar increases of *miR-200c *expression were seen in KP-3 and PANC-1 cell lines (data not shown).

### Upregulation of *miR-200c *enhanced the levels of *E-cadherin *mRNA in pancreatic cancer cells

We also investigated the levels of *E-cadherin *mRNA in the precursor and control groups. As shown in Figure 3b, the SUIT-2 precursor group, which expressed *miR-200c *at levels 38-fold higher than the control group, showed 3.9-fold higher *E-cadherin *mRNA levels compared to the control group 24 h after transfection.

### Upregulation of *miR-200c *stimulated proliferation in cancer cells

**Figure 4:**
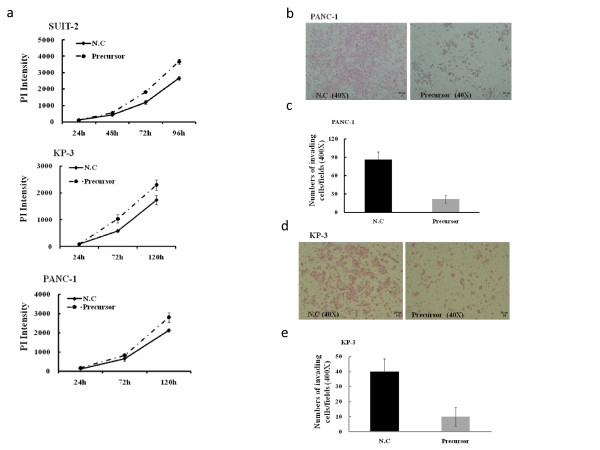
**Upregulation of *miR-200c *stimulated proliferation and inhibited invasion**. a. the upregulation of mature *miR-200c *expression enhanced the cell proliferation in an upregulation rate-dependent manner in SUIT-2 (upper), KP-3 (middle), and PANC-1 (bottom) cells. b and d. Pictures of invading cells (PANC-1 and KP-3, respectively). Original magnification, 10 ×. c and e. The number of invading cells (PANC-1 and KP-3, respectively) in five randomly selected fields observed under a light microscope (H&E staining, magnification, 400 ×). Error bars represent SD.

After confirmation of the upregulation of *miR-200c *in pancreatic cancer cells, we monitored changes in cell proliferation in PANC-1, SUIT-2, and KP-3 cell lines. As shown in Figure 4a, the upregulation of mature *miR-200c *expression in the precursor group enhanced cell proliferation in an upregulation rate-dependent manner for 96 h after transfection in SUIT-2 cells (upper), and for 120 h in KP-3 (middle) and PANC-1 cells (bottom).

### Upregulation of *miR-200c *inhibited invasion of cancer cells

Next, we investigated the effect of upregulation of mature *miR-200c *expression on the invasive potential of pancreatic cancer cells. Representative microphotographs of cells invading through Matrigel-coated membranes 36 h after transfection are shown for the control and *miR-200c *precursor cells in the left and right panels of Figure 4b, respectively. The numbers of invading PANC-1 cells were significantly inhibited in an upregulation rate-dependent manner when cells were transfected with the *miR-200c *precursor (*p *< 0.001), and the number of cells invading in the precursor group was approximately 75% less than the number of cells invading in the control group (Figure 4c). Similar to the inhibition rate of the PANC-1 precursor group, the KP-3 precursor group also showed a significant inhibition of invasion compared to the control group, with the control group invasion rate inhibited in the precursor group by approximately 75% (Figure 4d, e).

### Quantitative analysis of *miR-200c *and *E-cadherin *mRNA levels in macro-dissected FFPE pancreatic cancer tissues

**Figure 5:**
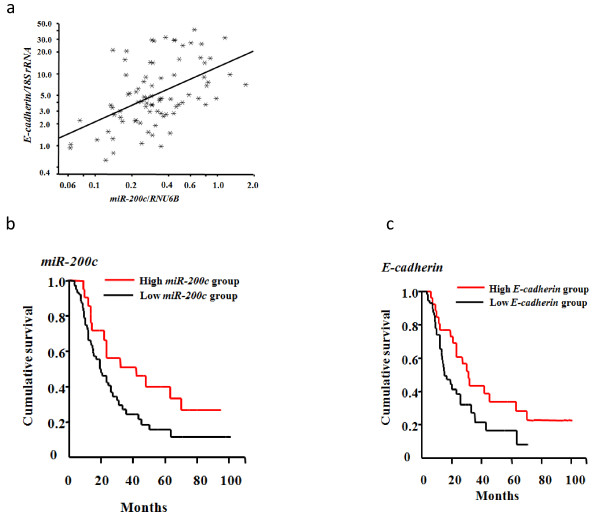
**The correlation between *miR-200c *and *E-cadherin *expression in macro-dissected FFPE pancreatic cancer tissues**. a. There was a significant correlation between *miR-200c *and *E-cadherin *mRNA levels in all macro-dissected pancreatic cancer tissues (Pearson's test *p *< 0.0001). b and c. Overall survival time after resection of pancreatic cancer with high *miR-200c *expression levels versus *low miR-200c *expression levels and with high *E-cadherin *mRNA expression levels versus low *E-cadherin *expression levels. Each sample was run in triplicate.

We measured *miR-200c *versus *E-cadherin *mRNA levels in macro-dissected FFPE samples from 99 patients who underwent pancreatic resection for pancreatic cancer at our institution from 1992 to 2007. The median *miR-200c *expression level in the macro-dissected pancreatic cancer samples was 0.30, and the median *E-cadherin *expression level was 4.41. Similar to the results from cultured cells, we also found that there was a significant correlation between *miR-200c *and *E-cadherin *mRNA levels in all macro-dissected pancreatic cancer tissues (Pearson's test *p *< 0.0001, Figure 5a).

### Univariate and multivariate analyses of *miR-200c *expression for survival time of patients with pancreatic cancer after curative resection

We classified the patients into two groups of high versus low *miR-200c *expression (cut-off value: 0.64; the partition was constructed by the overall survival time). The high and low *miR-200c *expression groups were composed of 21 and 78 cases, respectively. In univariate survival analyses based on the Cox proportional hazard model, the *miR-200c *levels and conventional prognostic factors, such as pT status (pT3 and pT4 versus pT1 and pT2), pN status (pN1 versus pN0), UICC stage (IV, III and IIB versus IIA, and versus IA and IB), tumor grade (G3 versus G1 and G2), R factor (R1 versus R0) and vessel invasion (positive versus negative), were investigated for overall survival (Table 2). We found that pT status, pN status, UICC stage, R factor and vessel invasion were significantly associated with a shorter overall survival (*p *< 0.001, *p *< 0.001, *p *= 0.002, *p *< 0.001, and *p *= 0.001, respectively). We also found that high *miR-200c *expression was associated with a longer overall survival (*p *= 0.03). The median survival time (MST) and 5-year survival rate were 42 months and 33.5% in the high *miR-200c *expression group, and 19 months and 11.2% in the low *miR-200c *expression group, respectively (Figure 5b). In multivariate survival analyses, we found that the overall survival time was significantly dependent on UICC stage (*p *= 0.01), R factor (*p *< 0.001), vessel invasion (*p *= 0.03) and high *miR-200c *expression (*p *= 0.02).

## Discussion

The present study shows, for the first time, the involvement of *miR-200c *in pancreatic cancer progression and prognosis. We have found that high *miR-200c *expression was related to low invasion ability, and that upregulation of *miR-200c *expression inhibited cell invasion and stimulated cell proliferation in pancreatic cancer cell lines. We also have found a significant correlation between *miR-200c *and *E-cadherin *expression, and that upregulation of *miR-200c *expression correlated with increased expression of *E-cadherin *in pancreatic cancer cell lines. This finding is consistent with previous reports investigating other cancers [14-19]. On the other hand, reduced expression of E-cadherin is regarded as a main molecular event in the dysfunction of the cell-cell adhesion system, triggering cancer invasion and metastasis [33,34]. Recently, Liu *et al*. revealed that E-cadherin stimulated cell proliferation at intermediate seeding densities, and Mees *et al*. revealed that metastasis suppressor gene EP300 was regulated by *miR-200c *in ductal adenocarcinomas of the pancreas [35,36]. These studies indicate that *miR-200c *plays a key role in the enhancement of proliferation and inhibition of invasion in pancreatic cancer via regulation of E-cadherin. Such inconsistent function is similar to gamma-interferon, which can inhibit tumor growth and enhance metastasis in a TS/A mammary adenocarcinoma model [37].

Furthermore, univariate and multivariate analyses of 99 macro-dissected FFPE pancreatic cancer samples revealed that high *miR-200c *expression was associated with a better prognosis. *E-cadherin *is considered as a prognosis factor in some cancers [38], and we also found that high *E-cadherin *expression was associated with a better prognosis in univariate analyses of macro-dissected FFPE pancreatic cancer samples but not in multivariate analyses (data not shown). These findings indicate that E-cadherin can be used as a prognosis factor by immunohistochemistry to detect E-cadherin protein or by qRT-PCR to measure *E-cadherin *mRNA levels. However, it is difficult to generate a highly specific E-cadherin protein antibody or specific *E-cadherin *mRNA primers, especially when using fragmented RNA from FFPE samples. miRNAs are small RNAs of 14 -24 nucleotides and are more stable than mRNA from FFPE samples [39] and the technologies of miRNA extraction and of qRT-PCR can be controlled more easily than those for mRNAs. Taken together, these findings suggest that *miR-200c *can be a better independent prognosis factor than *E-cadherin *mRNA in univariate or multivariate analyses, while the latter can be used as a prognosis factor in univariate analyses only. Furthermore, Mitchell, *et al*. reported that circulating miRNAs are stable blood-based markers for cancer detection [40], suggesting that quantifying the levels of *miR-200c *from patients' pancreatic juice or blood may provide an important marker for indicating the suitability for surgery.

In conclusion, our results have revealed that high levels of *miR-200c *expression inhibit cancer invasion and stimulate cancer cell proliferation, possibly via up-regulation of *E-cadherin*, and that high levels of *miR-200c *expression correlate with better survival of patients with curative resection of pancreatic cancer. We believe that research into *miR-200c *may bring about new opportunities for the development of drugs and therapeutic strategies for the treatment of pancreatic cancer. On the other hand, the *miR-200 *family members, like *miR-200a/b/c*, *miR-141*, and *miR-429*, have similar, but not identical functions ([19,20] and [36]). We believe that it is necessary to investigate the other family members to complete the picture regarding the *miR-200 *family and pancreatic cancer.

## Competing interests

The authors declare that they have no competing interests.

## Authors' contributions

Conception and design: JY, KO, KM, and MT; analysis and interpretation: JY, TK, and KN; data collection: HF; writing the article: JY; critical revision of the article: KO, KM, NS, and MT; final approval of the article: JY and MT; statistical analysis: JY, KO, and KN; overall responsibility: KO and KM. All authors read and approved the final manuscript.
